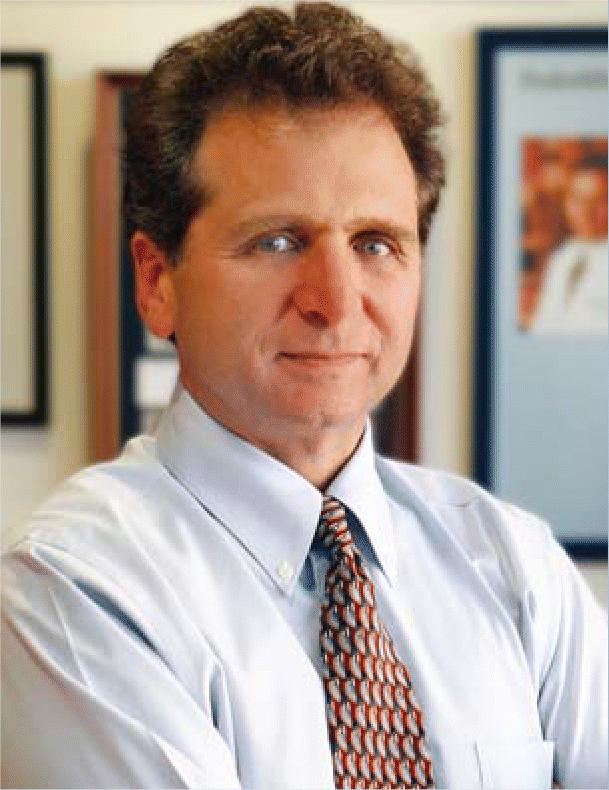# New Pathways to Disease Prevention

**Published:** 2007-02

**Authors:** David A. Schwartz

**Affiliations:** Director, NIEHS and NTP, E-mail: david.schwartz@niehs.nih.gov

As scientists, communication—not only with those outside our area of expertise but also with those within our scientific sphere—is an important responsibility, though often an ongoing challenge. As NIEHS director, I increasingly realize that, as the writer André Gide said, “The most important things to say are those which often I did not think necessary for me to say—because they were too obvious.” In this case, the thing that I thought was obvious, but now believe needs to be clearly reiterated and more fully described, is the focus of my vision and the work of this institute on the prevention of human disease.

In retrospect, I can see how discussions of my role as a physician and my strong emphasis on the value of clinical and translational research to the environmental health sciences may have created a misperception that my sole interest is clinical disease. People tend to talk most often and loudly about those things which they are passionate about. In reality, it is my role as a physician that has enabled me to recognize that clinical research and fundamental research in disease prevention are not separate paths, but rather intricately and necessarily connected approaches to achieving health. Though I have witnessed the enormous value of clinical and translational research to alleviate suffering from disease, I deeply believe that a far greater impact on human health can be attained by harnessing the power of this research toward understanding the etiology of disease and focusing this knowledge on preventing illness and death. It is this concept that is embodied in the NIEHS vision, which, as stated in our Strategic Plan, is “to prevent disease and improve human health by using environmental sciences to understand human biology and human disease.”

The greatest impacts on global environmental health will be made through breakthroughs in our basic understanding of the causes and mechanisms of disease.

A changing concept of disease prevention may also contribute to confusion on this issue. There are different approaches to preventing environmentally mediated disease. The first is to prevent all exposure to harmful environmental agents. The historical work of this institute has had a profound effect in this area by identifying agents such as metals, chemicals, and pollutants, and informing public policies to protect against them. But technology, time, money, and behavioral and societal factors are all real and constant hindrances to these efforts. A second approach is to identify those populations who may be most susceptible to environmental insults due to factors such as age, genetics, and health status, and then prevent exposures to these populations discretely. Ongoing studies funded by the NIEHS are focused on examining just such populations, but many of the same limitations of the first approach also apply here. A third approach, intervention between exposure and disease, is the most ambitious scientifically and a natural progression of the field of environmental health. Ambitious because we are pushing the bounds of knowledge and technology in our quest for understanding the biological effects of environmental exposures on mechanisms of human disease, and a natural progression because of the enormous potential of this approach to change the way we view disease and our ability to prevent it.

In reality, all three approaches complement each other and will continue to be part of our arsenal of disease prevention tools. Examples from the NIEHS research portfolio illustrate various ways in which we are exploring disease prevention strategies. A project in a low-income community in Washington State is implementing multilevel strategies including innovative housing for people with asthma, clinical asthma care, in-home education, and resident empowerment, along with scientific assessment and analysis, to decrease asthma morbidity and improve the built environment of public housing. Another project is developing bioinformatics tools to identify polymorphic promoter response elements in candidate environmental response genes such as *p53* to help determine the limits of human variability in risk assessment and thereby aid in disease prevention. The newly instituted Exposure Biology Program will move the science toward intervention between exposure and disease by providing sensitive biomarkers of exposure, susceptibility, and early biological response.

It is my belief that in the near future, the greatest impacts on global environmental health in terms of reduced morbidity and mortality will be made through breakthroughs in our basic understanding of the causes and mechanisms of disease. As we continue to develop new directions and priorities for the NIEHS, I hope that it will become ever more clear that in no way are we abandoning our long-held goal of disease prevention, but rather we are expanding our sights and exploring new pathways for understanding human biology and disease, always with this critical goal in mind.

## Figures and Tables

**Figure:**